# Supplementary material for: A Molecular Analysis of the Aminopeptidase P-Related Domain of PID-5 from Caenorhabditis elegans
Source: Biomolecules. 2023 Jul 14;13(7):1132. doi: 10.3390/biom13071132 (PMC10377022; doi:10.3390/biom13071132)
Supplement: Supplementary file 1 [file biomolecules-13-01132-s001.zip › biomolecules-2495387-supplementary.pdf]

Supplementary material

Supplementary Table S1. Detailed apstatin interactions of APP-1 structures and PID-5 APP-RD (predicted, AlphaFold2 DB: Q9GUI6). Reference literature shown in parentheses: *Ce*APP-1 [24], *Pf*APP-1 [8,26] and *Ec*APP-1 [22,24].

Protein	Residues	Apstatin Interactions	
		APP-1	Apstatin
*Ce*APP-1	Arg78	NH1<br>NH1	O3<br>O4
	His392	NE2	O3
	Asp413	OD2<br>OD1<br>OD2	O1<br>N1<br>N1
	His487	NE2	O2
	His496	NE2	O2
	Glu522	OE1<br>OE1<br>OE2	O1<br>N2<br>O2
	Glu536	OE1<br>OE2	O1<br>O1
	Zn^{2+}_A	-	O1
	Zn^{2+}_B	-	N1<br>O1
*Pf*APP-1	Asp581	OD1	N1
	His653	NE2	O2
	Glu676	OE1	N2
	Mn^{2+}_A	-	O1<br>O2
	Mn^{2+}_B	-	O1
*Ec*APP-1	His243	NE2	O3
	Asp260	OD1	N1
	His361	NE2	O2
	Glu383	OE1	O1
	Mn^{2+}_A	-	O1
	Mn^{2+}_B	-	N1<br>O1
Predicted PID-5 APP-RD	Gln857	NE2	O1
	Glu929	OE1<br>OE1	N4<br>N5
	His932	NE2	N1
	Glu950	OE1	N5
	Arg979	NH1<br>NH2<br>NH2	O1<br>O1<br>O2
	Zn^{2+}_A	-	N1<br>O1

Supplementary Table S2. Complete interactions of *Ce*APP-1 with apstatin (PDB code: 4S2T). Equivalent residues in PID-5 APP-RD (AlphaFold2 DB: Q9GUI6) are shown on the same row and potential interactions of these residues with apstatin, in the binding pose from the highest scoring GOLD ligand docking, are shown.

***Ce*APP-1 residues**	**Apstatin interactions**	**PID-5 APP-RD residues**	**Potential apstatin interactions**
Tyr43	Hydrophobic	Leu492	Hydrophobic
Asp76	Hydrophobic	Asp525	Hydrophobic
Arg78	2 H-bonds side chain NH1 to apstatin O3 and O4	Gly527	Hydrophobic
Phe378	Hydrophobic	Gln825	Hydrophobic
Asp379	Hydrophobic	Pro826	Hydrophobic
Ile381	Hydrophobic	Ile828	Hydrophobic
His392	H-bond side chain NE2 to apstatin O3	Ala839	Hydrophobic
Asp413	3 H-bonds to apstatin and metal coordination OD1 to Zn^{2+}_B	Gln857	H-bond side chain NE2 to apstatin O1
Asp424	Metal coordination side chain OD1 to Zn^{2+}_B and OD2 to Zn^{2+}_A	Asn868	Lost metal coordination
His483	Hydrophobic	His928	Hydrophobic
Gly484	Hydrophobic	Glu929	2 H-bonds side chain OE1 to apstatin N4 and N5
Gly486	Hydrophobic	Gly931	Hydrophobic
His487	H-bond side chain NE2 to apstatin O2 and Zn^{2+}_A	His932	H-bond side chain NE2 to apstatin N1 and Zn^{2+}_A
His496	H-bond side chain NE2 to apstatin O2	Arg941	Hydrophobic
Arg505	Hydrophobic	Glu950	H-bond side chain OE1 to apstatin N5
Glu522	3 H-bonds to apstatin and metal coordination OE2 to Zn^{2+}_A	Glu967	Metal coordination side chain OE2 to Zn^{2+}_A
Arg534	Hydrophobic	Arg979	3 H-bonds side chain NH1 to apstatin O1, and NH2 to O1 and O2
Glu536	2 H-bonds to apstatin and metal coordination OE1 to Zn^{2+}_B and OE2 to Zn^{2+}_A	Gly981	Lost metal coordination
Zn^{2+}_A	Coordination to apstatin O1	Zn^{2+}_A	Coordination to apstatin N1 and O1
Zn^{2+}_B	Coordination to apstatin O1 and N1	-	-
